# Novel Bionectriaceae (Hypocreales) taxa associated with ferns in southwestern China: morpho-phylogenetic description of two new species and one new host record

**DOI:** 10.3897/mycokeys.136.185538

**Published:** 2026-06-29

**Authors:** Jing-Yi Zhang, Ya-Ru Sun, Gui-Li Zhao, Shu-Qiong Xie, Yong-Zhong Lu, Yuan-Pin Xiao, Ning-Guo Liu

**Affiliations:** 1 School of Food and Pharmaceutical Engineering, Guizhou Institute of Technology, Guiyang 550025, China Guizhou Key Laboratory of Agricultural Microbiology, Guizhou Academy of Agricultural Sciences Guiyang China; 2 Department of Light Industry and Chemical Engineering, Guizhou Light Industry Polytechnic University, Guiyang 550025, China School of Food and Pharmaceutical Engineering, Guizhou Institute of Technology Guiyang China; 3 Guizhou Key Laboratory of Agricultural Microbiology, Guizhou Academy of Agricultural Sciences, Guiyang 550009, China School of Chemical Engineering, Guizhou Institute of Technology Guiyang China; 4 School of Chemical Engineering, Guizhou Institute of Technology, Guiyang 550025, China Department of Light Industry and Chemical Engineering, Guizhou Light Industry Polytechnic University Guiyang China

**Keywords:** 2 new taxa, phylogeny, Sordariomycetes, taxonomy

## Abstract

In a survey of microfungi associated with ferns in southwestern China, two novel taxa and a new host record of the family Bionectriaceae, *viz*., *Musananaesporium pronephrii***sp. nov**., *Waltergamsia yunnanensis***sp. nov**. and *Protocreopsis alba*, were collected and introduced via morphological observations and multi-gene phylogenetic analyses of combined LSU, ITS, *tef*1-α, and *rpb*2 sequence dataset. These three species share key morphological features, including hyaline, simple conidiophores and phialidic conidiogenous cells that produce hyaline, slimy conidia. This study expands the known diversity of Bionectriaceae and represents the first record of *Musananaesporium*, *Protocreopsis*, and *Waltergamsia* species associated with ferns.

## Introduction

Bionectriaceae (Hypocreales) is a cosmopolitan family that occurs primarily in terrestrial and freshwater habitats (Zhao et al. 2025). Members of this family can be saprobic, endophytic, parasitic, hyperparasitic, or lichenicolous (Maharachchikumbura et al. 2016; Hou et al. 2023; Zhao et al. 2025). Species within Bionectriaceae are widely utilized in agricultural, pharmaceutical, and commercial applications. For example, some *Clonostachys* species are used as natural biopesticides in agriculture (Reyes-Estebanez and Mendoza-de Gives 2025) and the secondary metabolites isolated from *Acremonium* species exhibit diverse biolo­gical activities, including antimicrobial, cytotoxic, and antitumor properties (Tian et al. 2017). Currently, the family comprises 50 recognized genera (Zhao et al. 2025).

*Protocreopsis* was proposed by Doi (1977) based on the type species *P. zingibericola*, along with *P. palmicola*. The sexual morph of *Protocreopsis* is cha­racterized by perithecial, papillate or apapillate, ostiolate ascomata surrounded by white to tan hyphae, clavate to fusoid, unitunicate asci, and bi- to pluriseriate, ellipsoid to fusoid, 1-septate, hyaline, typically striate ascospores. The asexual morph is hyphomycetous and acremonium-like, with monophialidic, usually unbranched, septate conidiophores, and ellipsoid or fusiform, (sub) cylindrical, oblong, aseptate, hyaline, slimy conidia (Doi 1977; Hou et al. 2023; Zhao et al. 2025). The genus has undergone several updated revisions based on molecular data. As a result, some species formerly placed in *Protocreopsis* have been transferred to related genera such as *Clavatomyces*, *Lasionectria*, and *Nectria* due to polyphyly in phylogenetic analyses (Forin et al. 2020; Zhao et al. 2025). Currently, 26 species are accepted in this genus.

*Musananaesporium* is a monotypic genus introduced by Hou et al. (2023) to accommodate *Acremonium tectonae*, isolated from a living leaf of *Tectona grandis* (Lamiaceae), based on morphological examination and phylogenetic inference. *Musananaesporium* is characterized by unbranched or basitonously branched conidiophores, mono- or polyphialidic conidiophores, and ellipsoidal, cylindrical, obclavate and fusoid, hyaline, 1–4-septate conidia produced in slimy heads (Hou et al. 2023).

Hou et al. (2023) transferred eight *Acremoniumsensu lato* species and described four novel species within *Waltergamsia*. *Waltergamsia* is characterized by unbranched or poorly branched, septate, hyaline conidiophores, mono- or polyphialidic, lateral or terminal conidiogenous cells, and variously shaped, aseptate, hyaline or subhyaline conidia arranged in slimy heads or dry chains (Hou et al. 2023; Liu et al. 2025). Species of *Waltergamsia* are distributed worldwide and occur as saprobic, coprophilic, and soil-inhabiting fungi as well as species associated with humans (Hou et al. 2023; Liu et al. 2025).

In this study, three new collections belonging to Bionectriaceae were obtained during a survey of microfungi associated with ferns in southwestern China. Based on detailed morphological observations and multi-gene phylogenetic analyses, we introduce two novel species, *Musananaesporium pronephrii* and *Waltergamsia yunnanensis*, and a new host record of *Protocreopsis alba*, all of which are described and illustrated in detail.

## Materials and methods

### Specimen collection, examination and isolation

Samples of decaying stems of ferns were collected in Guizhou and Yunnan Provinces, China, in terrestrial habitats. The samples were packaged in envelopes and brought to the laboratory. The fruiting bodies on natural substrates were observed and photographed using a Nikon SMZ 745 dissecting microscope (Nikon, Tokyo, Japan). Micro-morphological characters were observed and photographed using a Nikon ECLIPSE Ni compound microscope (Nikon, Japan) equipped with a Canon 90D digital camera (Canon, Japan). The Adobe Photoshop CS6 Extended v. 13.0 software was used to make photo-plates. Measurements were done with the Tarosoft (R) Image Frame Work software.

Single spore isolations were used to obtain pure cultures. Germinated conidia were transferred to new potato dextrose agar (PDA) plates and incubated at room temperature for four weeks. The pure cultures obtained were deposited in the Kunming Institute of Botany Culture Collection (KUNCC), Kunming, China, and Guizhou Culture Collection, China (GZCC) Guiyang, China. Herbarium materials were deposited in the Herbarium of Cryptogams, Kunming Institute of Botany, Academia Sinica (HKAS), Kunming, China. The scientific names of the new species were registered in Fungal Names (https://nmdc.cn/fungalnames/registe). The registration details are: *Musananaesporium pronephrii* (FN 573155) and *Waltergamsia yunnanensis* (FN 573157).

### DNA extraction, PCR amplification and sequencing

Fresh fungal mycelium grown on PDA medium was scraped with a sterile scalpel. Genomic DNA was extracted from scraped mycelium using the Ezup Column Fungi Genomic DNA Purification Kit (Sangon Biotech, Shanghai, China), following the manufacturer’s instructions. Four gene regions were selected in this study: the 28S subunit rDNA (LSU), the internal transcribed spacers (ITS), the translation elongation factor 1-alpha (*tef*1-α), and the RNA polymerase second largest subunit (*rpb*2). Polymerase chain reaction (PCR) was carried out in a 25 μL reaction volume, consisting of 1 μL of DNA, 1 μL each of forward and reverse primers, and 22 μL of 1.1 × T3 Super PCR Mix (Qingke Biotech, Chongqing, China). The PCR thermal cycle program and primers are given in Table 1. Amplified products were purified and sequenced by Tsingke Biotechnology Co., Ltd. (Beijing, China).

**Table 1.** Primers and PCR procedures used in this study.

**Locus**	**Primers**		**PCR procedures**	**References**
	**Name**	**Sequence (5’-3’)**		
LSU	LR0R	ACCCGCTGAACTTAAGC	94 °C 3 min; 35 cycles of 94 °C 30 s, 52 °C 30 s, 72 °C 1 min; 72 °C 8 min; 4 °C on hold	Vilgalys and Hester (1990); White et al. (1990)
	LR5	TCCTGAGGGAAACTTCG		
ITS	ITS5	GGAAGTAAAAGTCGTAACAAGG		
	ITS4	TCCTCCGCTTATTGATATGC		
*tef*1-α	EF1-983F	GCYCCYGGHCAYCGTGAYTTYAT	94 °C 2 min; 36 cycles of 66 °C–56 °C (touchdown 9 cycles), 94 °C 30 sec, 56 °C 1 min, 72 °C 1 min; 72 °C 10 min; 4 °C on hold	Rehner and Buckley (2005)
	EF1-2218R	ATGACACCRACRGCRACRGTYTG		
*rpb*2	fRPB2-5F	GAYGAYMGWGATCAYTTYGG	94 °C 3 min; 40 cycles of 94 °C 20 sec, 55 °C 30 sec, 72 °C 1 min; 72 °C 10 min; 4 °C on hold	Liu et al. (1999)
	fRPB2-7cR	CCCATRGCTTGYTTRCCCAT		

### Phylogenetic analyses

BLASTn (https://blast.ncbi.nlm.nih.gov//Blast.cgi) was used to evaluate closely related strains to our new taxa. Related sequences were downloaded from GenBank according to published literature (Table 2). The multiple alignments were automatically performed by online MAFFT version 7 (https://mafft.cbrc.jp/alignment/server/index.html). Trimal v1.2 (Capella-Gutierrez et al. 2009) was used to remove ambiguously aligned regions and uninformative positions with gappyout option. Four gene regions were combined using SequenceMatrix 1.7.8 (Vaidya et al. 2011). Alignments were checked visually using AliView (Larsson 2014). Sequences derived in this study were deposited in GenBank (Table 2).

**Table 2.** Taxa used in the phylogenetic analysis with the corresponding GenBank accession numbers. The newly generated strains are indicated in bold. N/A: Not available. T: Ex-type strain.

**Organism**	**Strain**	**ITS**	**LSU**	***rpb*2**	***tef*1-α**
*Amphichorda cavernicola*	CGMCC 3.19571 T	MK329056	MK328961	N/A	MK335997
*Amphichorda felina*	CBS 250.34	MH855498	OQ943167	N/A	OQ954490
*Amphichorda felina*	CBS 648.66	OQ942930	MH870575	N/A	OQ954491
*Clavatomyces palmarum*	HKAS 115709 T	NR_200711	NG_245588	N/A	PP761017
*Clonostachys buxicola*	CBS 102419 T	OQ910544	OQ910903	OQ927622	OQ944558
*Clonostachys rhinolophicola*	KUMCC 21-0439 T	ON426840	N/A	N/A	N/A
*Hydropisphaera cyatheae*	CBS 575.76	OQ429665	OQ055571	OQ454062	OQ470972
*Hydropisphaera peziza*	CBS 399.66	PV272846	PV273060	PV273407	PV273621
*Lasionectriella arenuloides*	CBS 576.76 T	OQ429696	OQ055601	OQ560701	OQ471006
*Lasionectriella rubioi*	CBS 132543	PV272834	PV273049	PV273396	PV273611
*Lasionectriella rubioi*	CBS 140157 T	OQ429699	KU593581	OQ454099	OQ471009
*Lasionectriopsis dentifera*	CBS 574.76 T	KY607540	KY607555	OQ454100	OQ471010
*Lasionectriopsis germanica*	CBS 143538 T	OQ429701	MK276528	OQ454102	OQ471012
*Lasionectriopsis germanica*	CBS 113762	PV272825	PV273040	PV273387	PV273602
*Monohydropisphaera fusigera*	CBS 124147 T	OQ429713	OQ055614	OQ454114	OQ471024
*Musananaesporium pronephrii*	KUNCC 23-13971 T	PX640680	PX640683	PX730066	PX730064
*Musananaesporium tectonae*	CBS 725.87 T	OQ429714	OQ055615	OQ454115	OQ471025
*Ochronectria calami*	CBS 134535	OQ429755	OQ055654	OQ560703	OQ471080
*Ochronectria thailandica*	MFLUCC 15-0140 T	KU564071	KU564069	N/A	N/A
*Paracylindrocarpon aloicola*	CBS 335.77	OQ429764	OQ055663	OQ454176	OQ471091
*Paracylindrocarpon aloicola*	CBS 141300 T	KX228277	KX228328	OQ454174	OQ471089
*Paracylindrocarpon foliicola*	CBS 140758 T	OQ429765	KX986914	OQ454177	OQ471092
*Parageonectria arachispora*	CBS 118.87 T	PV272843	PV273057	N/A	N/A
*Paragliomastix luzulae*	CBS 494.67	OR050512	OR052112	OQ454184	OQ471101
*Paragliomastix rosea*	CBS 277.80A T	OQ429775	OQ055673	OQ454186	OQ471103
*Protocreopsis alba*	KUNCC 23-14072	PX640679	PX640682	PX574981	PX574982
*Protocreopsis alba*	GMBC5357 T	PV933619	PV933637	PX373362	PX392344
*Protocreopsis alba*	GMBC5358	PV933620	PV933638	PX373363	PX392345
*Protocreopsis caricicola*	CBS 110505	PV272810	PV273026	PV273386	PV273588
*Protocreopsis caricicola*	CBS 140572 T	OQ429800	OQ055696	OQ451836	OQ471126
*Protocreopsis chlamydospora*	CBS 144254 T	PV272818	PV273033	N/A	PV273596
*Protocreopsis chlamydospora*	CBS 141859	PV272819	PV273034	N/A	N/A
*Protocreopsis euphorbiae*	CPC 38896 T	OK664700	OK663739	N/A	OQ471127
*Protocreopsis finnmarkica*	CBS 147428 T	OQ429803	OQ055699	N/A	OQ471130
*Protocreopsis finnmarkica*	CBS 147427	OQ429801	OQ055697	N/A	OQ471128
*Protocreopsis freycinetiae*	CBS 573.76 T	OQ429804	OR052113	OQ451837	OQ471132
*Protocreopsis gallica*	CBS 135079 T	PV272820	PV273035	N/A	PV273597
*Protocreopsis gallica*	CBS 141243	PV272821	PV273036	N/A	PV273598
*Protocreopsis globulosa*	KRAM-L 75079 T	NR_200883	PQ284592	N/A	PQ301172
*Protocreopsis loweniae*	Rodriguez-Flakus 4000 T	OR116443	OR133232	N/A	N/A
*Protocreopsis pertusa*	CBS 568.76	OQ429805	OQ430068	OQ454211	OQ471133
*Protocreopsis phormiicola*	CBS 567.76 T	OQ429806	OQ430069	N/A	OQ471134
*Protocreopsis physciae*	CBS 149678	NR_199758	NG_245639	N/A	N/A
*Protocreopsis rutila*	CBS 229.70	OQ429813	OQ430076	N/A	OQ471141
*Protocreopsis rutila*	CBS 396.66 T	OQ429814	OQ430077	N/A	OQ471142
*Protocreopsis vulpina*	CBS 565.76	PV272817	N/A	N/A	PV273595
*Pseudoacremonium sacchari*	CBS 137990 T	KJ869144	KJ869201	OQ454215	OQ471146
*Roumegueriella echinulata*	CBS 276.59 T	PV272862	PV273075	PV273423	PV273637
*Roumegueriella rufula*	CBS 346.85	OQ429827	OQ430088	DQ522461	OQ471156
*Selinia pulchra*	A.R. 2812	HM484859	GQ505992	N/A	HM484841
*Sesquicillium candelabrum*	CBS 119045 T	OQ911267	OQ911328	OQ914823	OQ944503
*Sesquicillium cavernum*	CV00218 T	OP856535	OP856525	N/A	OQ116931
*Smyrniomyces setaceus*	CBS 130334 T	PV272878	PV273088	PV273439	PV273652
*Stephanonectria chromolaenae*	MFLUCC 18-0589 T	ON230051	ON230059	N/A	N/A
*Stephanonectria keithii*	CBS 100007	OQ429871	OQ430120	OQ454269	OQ471202
*Tilachlidium brachiatum*	CBS 505.67	OQ429882	OQ430134	OQ454283	OQ471214
*Tilachlidium brachiatum*	CBS 363.97	KM231838	KM231719	KM232414	N/A
*Urticomyces pseudoarenulus*	CBS 128931	PV272882	PV273092	PV273443	PV273656
*Urticomyces pseudoarenulus*	AG09162	N/A	MT006266	N/A	N/A
*Verruciconidia erythroxyli*	CBS 728.87 T	OQ429910	OQ430161	OQ454307	OQ471240
*Verruciconidia verruculosa*	CBS 989.69 T	OQ429933	OQ430184	OQ454330	OQ471263
*Verrucostoma freycinetiae*	MAFF 240100 T	HM484866	GQ506013	N/A	HM484853
*Verrucostoma martinicense*	CBS 138731 T	OQ429934	OR052121	OQ454331	OQ471264
*Waltergamsia alkalina*	CBS 741.94 T	NR_189505.1	NG_242079.1	OQ454332.1	OQ471265.1
*Waltergamsia catenata*	CBS 102462 T	OQ429950	OQ430200	OQ454350	OQ471283
*Waltergamsia citrina*	CBS 384.96 T	NR_154670.1	NG_242080.1	OQ454333.1	OQ471266.1
*Waltergamsia dimorphospora*	CBS 139050 T	NR_189804.1	NG_228777.1	OQ454335.1	OQ471268.1
*Waltergamsia epimycota*	CBS 562.86	OQ429939	OQ430189	OQ454337	OQ471270
*Waltergamsia fusidioides*	CBS 840.68 T	FN706542	HQ232039	OQ454339	OQ471272
*Waltergamsia harwoodiae*	MST FP3578 T	PQ607744.1	PQ607750.1	PQ566643.1	PQ566644.1
*Waltergamsia hennebertii*	CBS 768.69 T	OQ429942	OQ430192	OQ454342	OQ471275
*Waltergamsia mali*	ACCC 39305 T	MF987658	MF993114	N/A	N/A
*Waltergamsia maroccana*	CBS 512.82 T	NR_189506.1	NG_242081.1	OQ454343.1	OQ471276.1
*Waltergamsia mpumalangana*	CBS 150065 T	NR_197903.1	NG_243930.1	OR683729.1	OR683716.1
*Waltergamsia obpyriformis*	CBS 595.73 T	OQ429945	OQ430195	OQ454345	OQ471278
*Waltergamsia parva*	CBS 381.70A T	OQ429946	OQ430196	OQ454346	OQ471279
*Waltergamsia pilosa*	CBS 124.70 T	NR_163809.1	OQ430199.1	OQ454349.1	OQ471282.1
*Waltergamsia yunnanensis*	GZCC 23-0746 T	PX640678	PX640681	PX730065	PX730063
*Waltergamsia zeylanica*	CBS 735.73A	OQ429954	OQ430204	OQ454354	OQ471287

The single locus and combined analyses were carried out for maximum likelihood (ML) and Bayesian inference (BI). ML analysis was performed using RAxML-HPC v.8 on ACCESS (8.2.12) (Stamatakis 2014) in CIPRES Science Gateway (Miller et al. 2010), using the GTR+GAMMA model with 1,000 bootstrap repetitions. Maximum likelihood bootstrap values (ML-BS) equal or greater than 75% are marked near each node.

Bayesian inference was carried out in MrBayes 3.2.6 (Ronquist et al. 2012) using a Markov chain Monte Carlo (MCMC) algorithm. The best-fit substitution model GTR + I +G was decided for LSU, ITS, *tef*1-α, and *rpb*2 by MrModeltest 2.3 (Nylander 2008) under the Akaike Information Criterion (AIC). Two parallel runs of six simultaneous Markov chains were performed for 10,000,000 generations. Trees were sampled every 1,000^th^ generations. Burn-in phase was set at 25% and the remaining trees were used for calculating posterior probabilities (PP). PP values equal to or greater than 0.95 are marked near each node.

Trees were visualized with FigTree v1.4.4 (http://tree.bio.ed.ac.uk/software/figtree), and the layout was edited using Adobe Illustrator CS6 software (Adobe Systems, USA).

## Results

### Phylogenetic analyses

**Figure 1.**
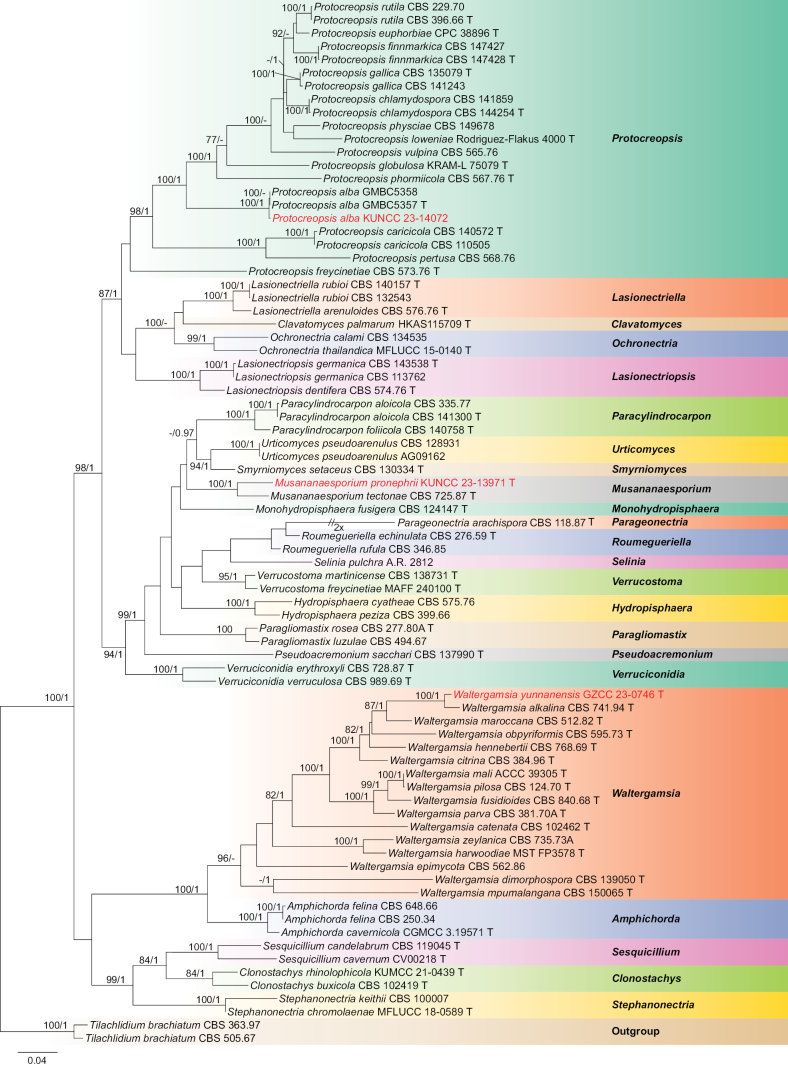
ML tree based on the combined LSU-ITS-*tef*1-α-*rpb*2 sequences. Bootstrap support values for ML greater than 75% and PP greater than 0.95 are given near nodes as ML-BS/PP. The tree is rooted with *Tilachlidium brachiatum* (CBS 363.97) and *T. brachiatum* (CBS 505.67). Abbreviation “T” denote ex-type strains. The new taxa are indicated in red.

The combined LSU, ITS, *tef*1-α, and *rpb*2 sequence matrix comprised 67 taxa, including the three new collections and one outgroup taxon, *Tilachlidium brachiatum* represented by CBS 363.97 and CBS 505.67. The alignment comprised 2,838 characters (LSU: 1–772, ITS: 773–1,274, *tef*1-α: 1,275–2,085, *rpb*2: 2,086–2,838), including gaps. Single locus phylogenetic analyses were performed to compare the topology and clade stability. Maximum likelihood and Bayesian analyses of the combined dataset inferred largely similar phylogenetic reconstructions. The ML tree (-ln = 36738.679087) is shown in Fig. 1.

The multi-gene analysis (Fig. 1) showed that our new isolate KUNCC 23-14072 grouped with *Protocreopsis alba* (GMBC5358 and GMBC5357). *Musananaesporium pronephrii* (KUNCC 23-13971) formed a sister clade with *M. tectonae* (CBS 725.87) with 100% ML and 1 PP support. *Waltergamsia yunnanensis* (GZCC 23-0746) clustered with other *Waltergamsia* species and was sister to *W. alkalina* (CBS 741.94) with 100% ML and 1 PP support.

### Taxonomy

#### *Musananaesporium* L.W. Hou, et al., in Hou, et al., Stud. Mycol. 105: 72 (2023)

##### Musananaesporium pronephrii

Taxon classificationFungiHypocrealesBionectriaceae

Jing Y. Zhang, Y.Z. Lu & N.G. Liu sp. nov.

6198A7C1-5296-5647-8ECC-C07325E9CA2F

Fungal Names: FN 573155

Fig. 2

###### Etymology.

The epithet “*pronephrii*” refers to the host genus *Pronephrium*, from which the fungus was isolated.

**Figure 2.**
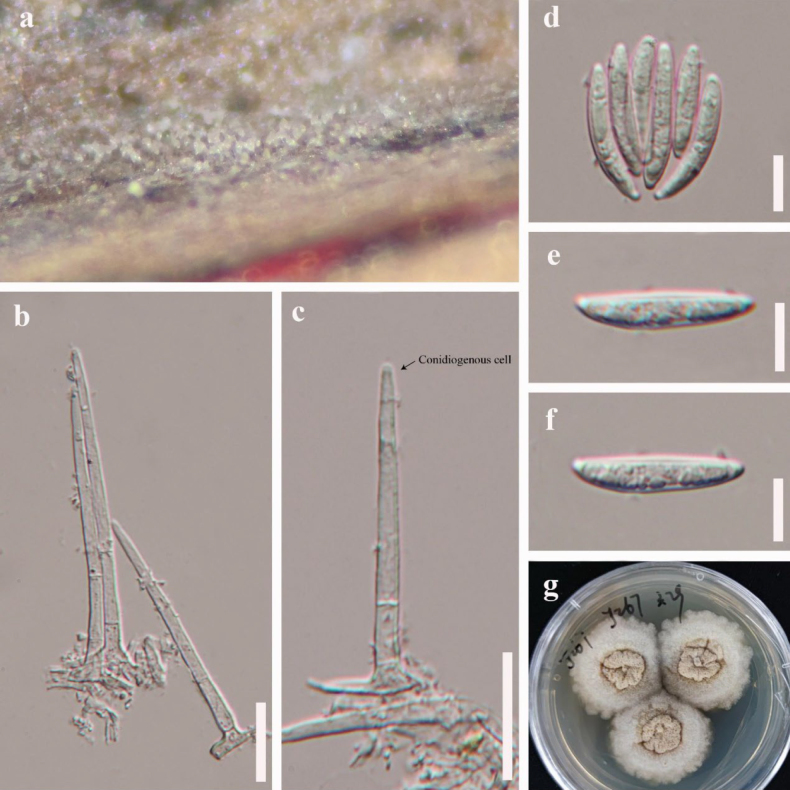
*Musananaesporium pronephrii* (HKAS 129760, holotype) **a**. Colonies on natural substratum; **b, c**. Conidiophores with conidiogenous cells; **d–f**. Conidia; **g**. Colonies on PDA. Scale bars: 20 μm (**b, c**); 10 μm (**d–f**).

###### Holotype.

HKAS 129760.

###### Description.

*Saprobic* on twigs of *Pronephrium penangianum*. ***Asexual morph***: Hyphomycetous. *Colonies* on natural substratum effuse, white, velvety. *Mycelium* immersed, composed of septate, branched, hyaline hyphae. *Conidiophores* 48–75 µm long, 3–5 µm wide at base, macronematous, mononematous, erect, straight or slightly curved, cylindrical, narrowed towards apex, unbranched, septate, hyaline, smooth-walled. *Conidiogenous cells* 6–15 μm long, monophialidic, integrated, terminal, determinate, cylindrical, hyaline, smooth-walled. *Conidia* 19–30 × 3.5–5 μm (x̄ = 24 × 4 μm, n = 30), arranged in a slimy head, narrowly fusoid, straight or slightly curved, hyaline, aseptate, thin- and smooth-walled. ***Sexual morph***: Undetermined.

###### Culture characteristics.

Conidia germinated on PDA within 12 hours at 25 °C. Germ tubes produced from both ends. Colony 25–30 mm diameter after 4 weeks at room temperature on PDA media. Mycelium superficial, irregularly circular, entire edge, wrinkled, dry, brown at center, white peripherally when viewed from above; pale brown when observed from below.

###### Material examined.

China • Guizhou Province, Qianxinan Bouyei and Miao Autonomous Prefecture, Anlong County, Xianheping National Forest Park, on dead stems of *Pronephrium penangianum* (Thelypteridaceae), 16 March 2022, J.Y. Zhang, J267 (HKAS 129760 ***holotype***, ex-type culture KUNCC 23-13971).

###### Notes.

*Musananaesporium pronephrii* is phylogenetically sister to *M. tectonae*, which was isolated from a living leaf of *Tectona grandis* (Lamiaceae) in Cuba (Hou et al. 2023) (Fig. 1). *Musananaesporium pronephrii* resembles *M. tectonae* in having hyaline conidiophores with inconspicuous collarettes and fusoid, hyaline conidia. However, the two species can be distinguished in that *M. pronephrii* possesses terminal monophialide and aseptate conidia, whereas *M. tectonae* features terminal and subterminal polyphialides, with conidia becoming 1–4-septate upon maturity. Additionally, the former is characterized by shorter conidiophores (48–75 µm vs. up to 268 μm in *M. tectonae*) and larger conidia (19–30 × 3.5–5 μm vs. 11.5–25.5 × 2.5–4.5 μm in *M. tectonae*) (Hou et al. 2023). In addition, *Musananaesporium pronephrii* (KUNCC 23-13971) differs from *M. tectonae* (CBS 725.87) by 26 bp (4.9%, without gap, 527 bp) in the ITS region, 9 bp (1.2%, without gap, 777 bp) in the LSU region, 49 bp (6.5%, without gap, 756 bp) in the *rpb*2 region, and 37 bp (4.6%, without gap, 808 bp) in the *tef*1-α region. Therefore, we introduce *Musananaesporium pronephrii* as a novel species herein.

#### *Protocreopsis* Yoshim. Doi, Kew Bull. 31(3): 551 (1977)

##### Protocreopsis alba

Taxon classificationFungiHypocrealesBionectriaceae

L.L. Liu, et al., Mycosphere 16(1): 4004 (2025)

1E635919-E15F-5E1C-96FA-C0367A9846A1

Fungal Names: FN 859987

Fig. 3

###### Holotype.

GMB5357.

**Figure 3.**
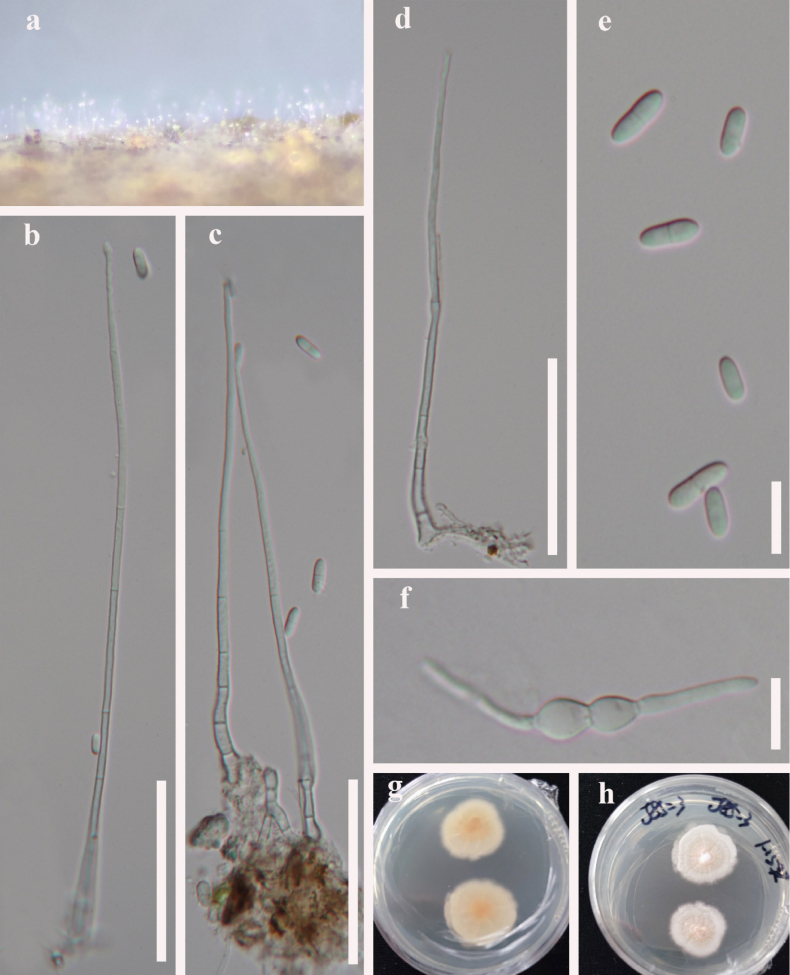
*Protocreopsis alba* (HKAS 129822, new host record) **a**. Colonies on natural substrate; **b–d**. Conidiophores with conidia; **e**. Conidia; **f**. Germinated conidium; **g, h**. Colonies on PDA (reverse view and top view). Scale bars: 50 μm (**b–d**); 10 μm (**e, f**).

###### Description.

*Saprobic* on frond stalks of *Angiopteris fokiensis*. ***Asexual morph***: Hyphomycetous. *Colonies* on natural substratum effuse, white, velvety. *Mycelium* immersed, composed of septate, branched, hyaline hyphae. *Conidiophores* up to 135 µm long, 3.5–5 µm wide at base, macronematous, mononematous, erect, straight or slightly curved, flexuous, cylindrical, tapering towards apex, unbranched, septate, hyaline, smooth-walled. *Conidiogenous cells* monophialidic, integrated, terminal, determinate, cylindrical, hyaline, smooth-walled. *Conidia* 6–10.5 × 2–3.5 μm (x̄ = 8 × 2.5 μm, n = 50), arranged in a slimy head, cylindrical, hyaline, 0–1-septate, slightly constricted at septum, thin- walled, smooth-walled. ***Sexual morph***: Undetermined.

###### Culture characteristics.

Conidia germinated on PDA within 12 hours at 25 °C. Germ tubes produced from both ends. Colony 20–25 mm diameter after 4 weeks at room temperature on PDA media. Mycelium superficial, circular, entire edge, dry, flat, white from above, pale yellow from below.

###### Material examined.

China • Guizhou Province, Guiyang City, Guiyang Medicinal Botanical Garden, on dead frond stalks of *Angiopteris fokiensis* (Marattiaceae), 13 December 2021, J.Y. Zhang, J85-3 (HKAS 129822, living culture KUNCC 23-14072).

###### Notes.

In a BLASTn search of NCBI GenBank, the closest matches to the LSU, ITS, *tef*1-α, and *rpb*2 sequences of our new isolate KUNCC 23-14072 were *Protocreopsis alba*, showing 100%, 100%, 99.89%, and 99.55% similarities, respectively. Morphologically, our isolate largely agrees with the original description of *P. alba*. Although slightly larger conidia (6–10.5 × 2–3.5 μm vs. 3.5–7.8 × 2–3.6 μm) and occasional septation observed in the present collection, these differences fall within the intraspecific variation of *P. alba*. Phylogenetic analyses further confirmed that our isolate clusters with two strains of *P. alba* (GMBC5358 and GMBC5357). *Protocreopsis alba* was originally introduced by Liu et al. (2025) from decaying wood in China. Accordingly, the present collection from dead frond stalks of *Angiopteris fokiensis* is identified as a new host record of *P. alba*.

#### *Waltergamsia* L.W. Hou, et al., Stud. Mycol. 105: 138 (2023)

##### Waltergamsia yunnanensis

Taxon classificationFungiHypocrealesBionectriaceae

Jing Y. Zhang, Y.Z. Lu & N.G. Liu sp. nov.

10F5BD25-A4F3-5004-8387-A6022AE3BB4B

Fungal Names: FN 573157

Fig. 4

###### Etymology.

The epithet “*yunnanensis*” refers to Yunnan Province, China, where the fungus was collected.

**Figure 4.**
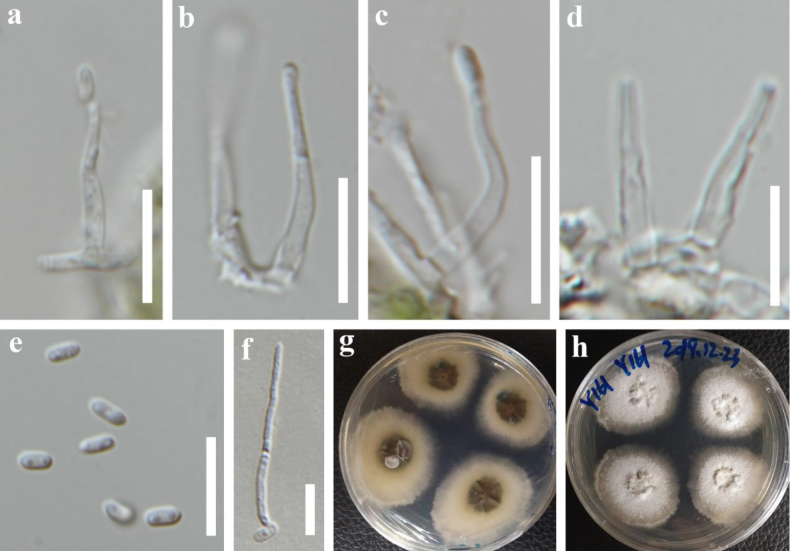
*Waltergamsia yunnanensis* (HKAS 129840, holotype) **a–d**. Conidiophores with conidia; **e**. Conidia; **f**. Germinated conidium; **g, h**. Colonies on PDA (reverse view and top view). Scale bars: 5 μm (**a–f**).

###### Holotype.

HKAS 129840.

###### Description.

*Saprobic* on twigs of unidentified fern. ***Asexual morph***: Hyphomycetous. *Colonies* on natural substratum effuse, white, velvety. *Mycelium* immersed, composed of septate, branched, hyaline hyphae. *Conidiophores* 5–7.5 μm long, about 1 μm wide at base, micronematous to semi-macronematous, mononematous, solitary, erect, straight or curved, cylindrical, tapering towards apex, unbranched, usually with a single basal septum, hyaline, smooth-walled. *Conidiogenous cells* monophialidic, integrated, terminal, determinate, cylindrical, hyaline, smooth-walled. *Conidia* 1.5–2 × 0.5–1 μm (x̄ = 1.7 × 0.7 μm, n = 50), arranged in a slimy head, oblong with rounded ends, or oval, hyaline, aseptate, with two guttulate, thin- and smooth-walled. ***Sexual morph***: Undetermined.

###### Culture characteristics.

Conidia germinated on PDA within 12 hours at 25 °C. Germ tubes produced from both ends. Colony 30 mm diameter after 4 weeks at room temperature on PDA media. Mycelium superficial, irregularly circular, entire edge, flat, dry, white from above, center brown, surrounded by white from below.

###### Material examined.

China • Yunnan Province, Xishuangbanna Dai Autonomous Prefecture, Mengla County, Menglun Town, Xishuangbanna Tropical Botanical Garden, Chinese Academy of Sciences (21°55'39"N, 101°15'15"E), on dead rachis of an unidentified fern, 16 November 2019, J.Y. Zhang, Y161 (HKAS 129840 ***holotype***, ex-type culture GZCC 23-0746).

###### Notes.

*Waltergamsia yunnanensis* is phylogenetically related to *W. alkalina* (CBS 741.94) and *W. maroccana* (CBS 512.82) (Fig. 1). Their morphological comparisons are shown in Table 3. In addition, *W. yunnanensis* differs from *W. alkalina* by 35 bp (including 8 gaps, 452 bp in total) in the ITS region, 3 bp (811 bp in total, no gaps) in the *tef*1-α region, and 10 bp (756 bp in total, no gaps) in the *rpb*2 region. Furthermore, it differs from *W. maroccana* by 62 bp (including 16 gaps, 494 bp in total) in the ITS region, 20 bp (811 bp in total, no gaps) in the *tef*1-α region, and 72 bp (756 bp in total, no gaps) in the *rpb*2 region. Therefore, we propose *Waltergamsia yunnanensis* as a novel species.

**Table 3.** Morphological comparisons of *W. alkalina*, *W. maroccana* and *W. yunnanensis*.

**Species**	**Conidiophores**	**Phialides (μm)**	**Conidia (μm)**	**References**
*W. alkalina*	hyaline, unbranched or poorly branched, bearing up to two phialides per node	13–32.8 × 1.3–2.5, terminal and lateral	3–5.4 × 1.2–1.8, cylindrical, hyaline, aseptate, eguttulate	Hou et al. (2023)
*W. maroccana*	unbranched or basitonously branched, bearing 1–2 levels with 2–4 divergent phialides per node	10.5–21.5 × 1–2.5, lateral	2.8–4 × 1.7–2.4, ovoid, ellipsoid, hyaline, aseptate, eguttulate	Hou et al. (2023)
*W. yunnanensis*	hyaline, unbranched bearing one phialide	5–7.5 × 1, terminal	1.5–2 × 0.5–1, oblong or oval, guttulate	This study

## Discussion

In this study, we introduce two new species and a new host record of fungi belonging to the family Bionectriaceae (Hypocreales). *Musananaesporium pronephrii* was found on dead stems of *Pronephrium penangianum* (Thelypteridaceae) and represents the second species of this genus. *Protocreopsis alba* was discovered on frond stalks of *Angiopteris fokiensis* (Marattiaceae). *Waltergamsia yunnanensis* was collected from the dead rachis of an unidentified fern. This new species is congeneric with the type of *Waltergamsia*, *W. fusidioides*, and is thus placed in that genus.

In our phylogenetic tree (Fig. 1), the monophyly of *Protocreopsis* is not well supported, which is consistent with previous studies. For example, *P. euphorbiae* grouped with two *Acremonium* species and *Pronectria robergei* in Crous et al. (2021). Additionally, *Protocreopsis* formed four polyphyletic clades in Visagie et al. (2024). Although *Protocreopsis* grouped together in Hou et al. (2023), its monophyly is poorly supported. More importantly, the current taxonomic classification of *Protocreopsis* within Bionectriaceae is based on subsequently introduced species, since its type species, *P. zingibericola* (*Pt. fusigera*), lacks molecular data. This absence of sequence data for the type species presents a fundamental obstacle to resolving the generic boundaries of *Protocreopsis*. Without reliable sequence data for *P. zingibericola*, it is challenging to accurately assess the phylogenetic relationships between this type species and other members of *Protocreopsis*, as well as its affinities with closely related genera in Bionectriaceae. Consequently, the circumscription of *Protocreopsis* remains uncertain, and any future taxonomic revision will require an epitypification of *P. zingibericola* with a sequenced specimen.

Although ferns represent an important link in vascular plant evolution and their associated fungi play irreplaceable roles in ecosystems, research on fern–fungal interactions, particularly regarding species diversity and taxonomy, remains markedly insufficient compared to seed plants (Zhang et al. 2025). Bionectriaceae is a species-rich family exhibiting diverse ecological niches. However, its occurrence on ferns remains relatively poorly documented, with only about 20 species reported (Zhang et al. 2025). Southwest China is a hotspot for fungal diversity research, where numerous novel species have been discovered in recent years (Yang et al. 2023; Liu et al. 2024; Ma et al. 2024; Lin et al. 2025; Sun et al. 2025; Xiao et al. 2025). Through in-depth surveys in this biodiversity hotspot, the present study enriches our understanding of the diversity of Bionectriaceae and, for the first time, establishes associations between *Musananaesporium*, *Waltergamsia* and ferns. The species described in this study adopt a saprobic lifestyle, colonizing dead fern tissues, which indicates that they likely play an important role in nutrient cycling and litter decomposition within ecosystems. However, considering the diverse lifestyles of Bionectriaceae species, including endophytic and parasitic (Zhao et al. 2023, 2025), these newly discovered taxa may also possess undiscovered endophytic or potential pathogenic capabilities.

## Supplementary Material

XML Treatment for Musananaesporium pronephrii

XML Treatment for Protocreopsis alba

XML Treatment for Waltergamsia yunnanensis

## Additional information

### Artificial Intelligence (AI) use

The authors accept full responsibility for the content of the manuscript, including the disclosure of any use of AI.

No AI tools were used in the preparation of this manuscript.

### Author ORCIDs

J.-Y. Zhang https://orcid.org/0000-0003-0606-6169

Y.-R. Sun https://orcid.org/0000-0001-5549-1028

G.-L. Zhao https://orcid.org/0000-0001-8370-4844

S.-Q. Xie https://orcid.org/0009-0006-8011-7680

Y.-Z. Lu https://orcid.org/0000-0002-1033-5782

Y.-P. Xiao https://orcid.org/0000-0003-1730-3545

N.-G. Liu https://orcid.org/0000-0002-9169-2350

### Data availability

All of the data that support the findings of this study are available in the main text.
